# High‐dose, short‐term corticosteroids for ARDS caused by COVID‐19: a case series

**DOI:** 10.1002/rcr2.596

**Published:** 2020-06-04

**Authors:** Clara So, Shosei Ro, Manabu Murakami, Ryosuke Imai, Torahiko Jinta

**Affiliations:** ^1^ Department of Pulmonary Medicine, Thoracic Center St. Luke's International Hospital Tokyo Japan

**Keywords:** ARDS, corticosteroid therapy, COVID‐19, mechanical ventilation

## Abstract

We report a case series of seven mechanically ventilated patients with acute respiratory distress syndrome (ARDS) caused by coronavirus disease (COVID‐19) who received early treatment with high‐dose, short‐term systemic corticosteroids to prevent cytokine overproduction. Of the seven patients, four were male and median age was 69 years. They were intubated within seven days after admission when their respiratory status rapidly worsened. At that time, we administered 1000 or 500 mg/day for three days of methylprednisolone intravenously, followed by 1 mg/kg and tapered off. The median duration for the total administration of corticosteroids was 13 days. This high‐dose, short‐term corticosteroid therapy enabled extubation of the patients within seven days. Many questions on the clinical management of COVID‐19 remain unanswered, and data on corticosteroid therapy as a choice of treatment are mixed. We present the clinical course of our cases, review the previous evidence, and discuss management.

## Graphical abstract

Many questions on the clinical management of coronavirus disease (COVID‐19) remain unanswered, and data on corticosteroid therapy as a choice of treatment are mixed. We present the clinical course of seven patients, review the previous evidence, and discuss management.

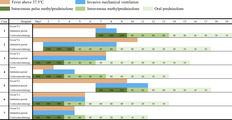

## Introduction

A novel coronavirus was identified in 2019 as the cause of a cluster of pneumonia cases in Wuhan, China. It has since rapidly spread, resulting in a pandemic. Although most patients have a favourable prognosis, some patients may have worse outcomes. Acute respiratory distress syndrome (ARDS) is the most common complication and occurs in 60–70% of patients admitted to the intensive care unit (ICU) [1, 2, 3]. Indeed, patients with severe illness may develop dyspnoea and hypoxaemia within one week of disease onset, and this may rapidly progress to ARDS [4]. It can progress to refractory respiratory failure and, in such cases, extracorporeal membrane oxygenation or other forms of whole‐body management may be considered as a rescue therapy [4]. In spite of intensive care and rescue therapy, the mortality from coronavirus disease (COVID‐19) appears to be driven by the presence of severe ARDS and is approximately 50% [2, 3, 5, 6]. There is an urgent need to find a way of preventing the progress of ARDS with COVID‐19 to improve mortality rate.

It has been theorized that ARDS‐like states, caused by cytokine overproduction [7], are implicated in the worsening of the illness. The underlying factors in disease progression remain elusive, and effective treatments have not been established [5]. Among the treatments, corticosteroid therapy has been employed in many cases, but its efficacy remains unclear; therefore, it has not been officially recommended [5, 8]. Moreover, there are some reports in which the effectiveness of corticosteroid therapy for COVID‐19 is discussed, but its dose and duration are not clear in most cases. We summarized the clinical course and treatment timeline of seven critically ill patients with ARDS with COVID‐19 who were mechanically ventilated and treated with high‐dose, short‐term corticosteroid therapy. None of the patients have been reported in another manuscript.

## Case Series

At St. Luke's International Hospital in Japan in March 2020, seven were admitted to the ICU with COVID‐19‐related ARDS when their oxygenation had been deteriorating rapidly. The diagnosis of COVID‐19 is made by detection of severe acute respiratory syndrome coronavirus 2 (SARS‐CoV‐2) RNA by real‐time polymerase chain reaction. High‐flow oxygen therapy such as high‐flow nasal cannula and non‐invasive positive pressure ventilation were not used because they could cause aerosol exposure and lead to the disposal of medical devices. The order of oxygen delivery device up was nasal cannulas, face masks, and reservoir masks. When PaO_2_/FiO_2_ ratio < 150 and a worsening trend continued despite the reservoir mask, we performed tracheal intubation. During ventilator management, appropriate amount of maintenance fluids was administered and diuresis was considered. Fluid balance was managed to minus balance as much as possible to prevent worsening of oxygenation. Respiratory settings were with a limit tidal volume of 6–8 mL/kg (predicted body weight), end‐inspiratory plateau pressure < 30 cmH_2_O, and driving pressure < 15 cmH_2_O. Positive end‐expiratory pressure was initially set at 8–10 cmH_2_O and increased to an upper limit of 14–15 cmH_2_O when pulmonary compliance was decreased and air content was low. No patient was taken with prone positioning.

**Figure 1:**
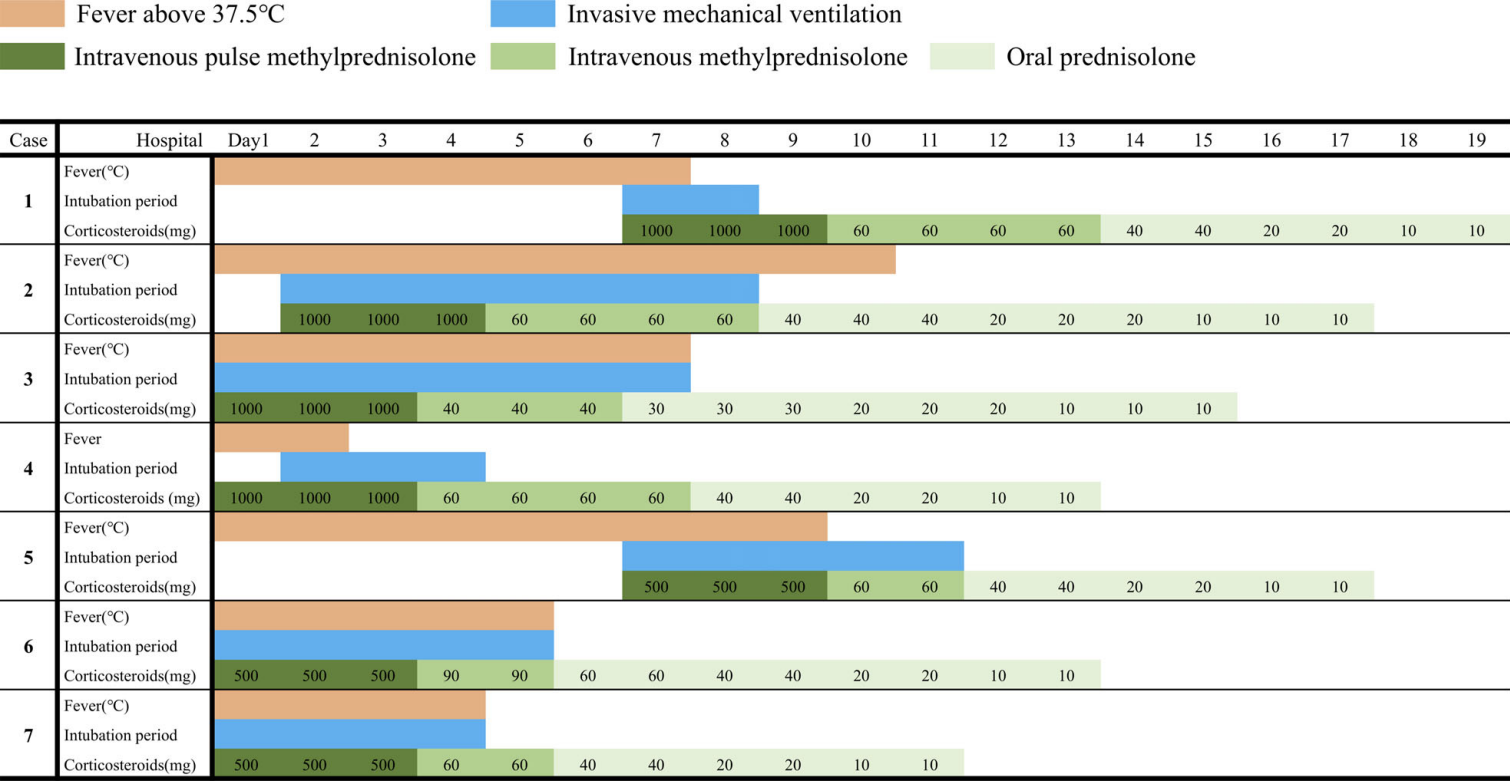
Timeline of disease course according to days from hospital admission. For each case, fever, intubation period, and the amount of corticosteroids were described. Regarding the fever, orange represents a fever of 37.5°C or higher. In terms of corticosteroids, dark green indicates intravenous methylprednisolone and light green indicates oral prednisolone.

**Figure 2:**
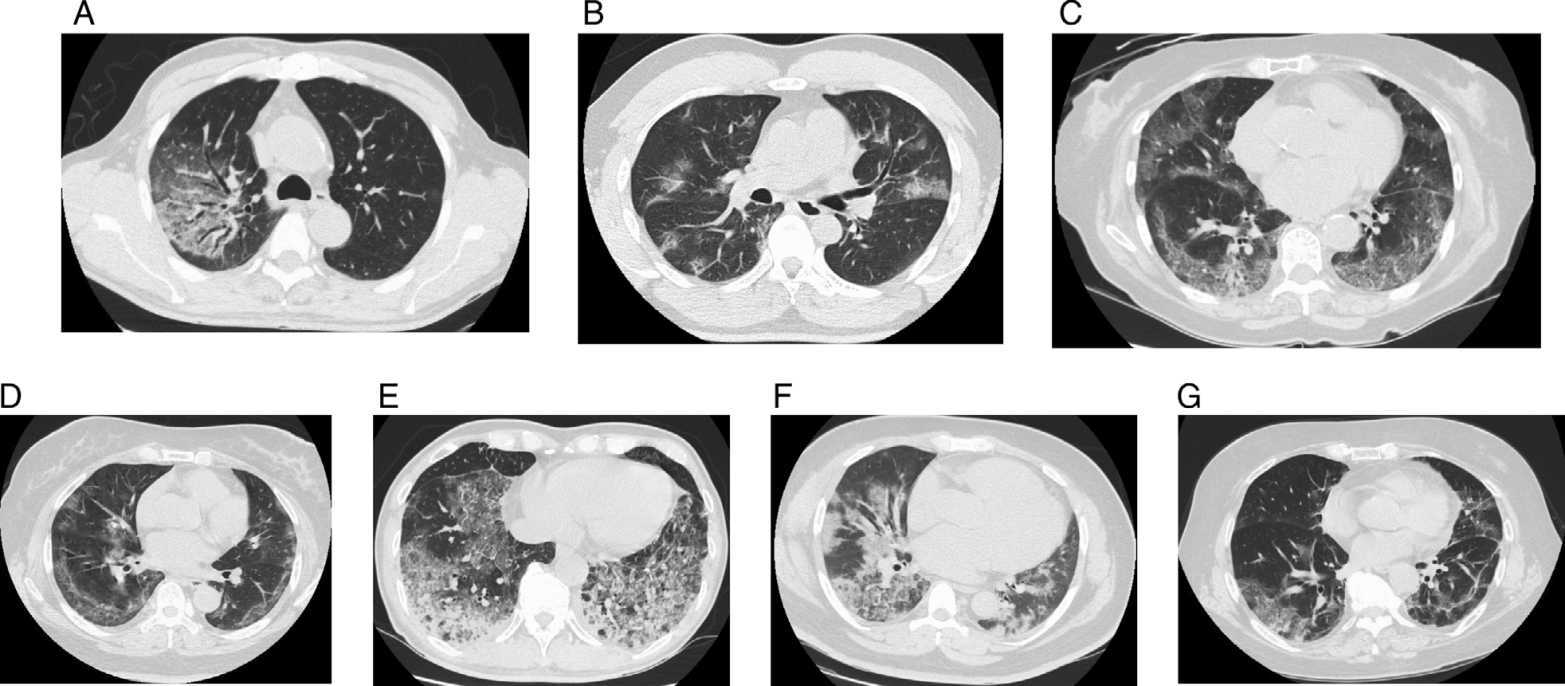
Unenhanced computed tomography images of seven patients on admission day. A–G corresponds to Case 1–7, respectively.

Four patients were male. The patients had a median age and body mass index of 69 years (range: 41–77) and 25.1, respectively. The median Brinkman index was 40, and comorbidities included asthma, chronic atrial fibrillation, diabetes, hypertension, and dyslipidaemia. The median time between fever onset and intubation was 11 days, the median PaO_2_/FiO_2_ ratio before intubation was 117, the median PaO_2_/FiO_2_ ratio after corticosteroid administration was 142, and the median fraction of inspired oxygen (FiO_2_) at 24 h after intubation was 0.4. At the exact moment when the patients' respiratory failure suddenly progressed, we started intravenous pulse methylprednisolone: 1000 or 500 mg/day of methylprednisolone for three days intravenously, followed by 1 mg/kg once daily, then tapered by 10 or 20 or 30 mg/day of prednisolone orally, finishing at 10 mg of prednisolone. The median duration of corticosteroids administration was 13 days. After the corticosteroid therapy, fever and oxygen demand decreased. The median C‐reactive protein levels before and after the therapy were 12.3 and 1.7 mg/dL, respectively. All patients were successfully extubated without reintubation and discharged from our hospital. The median time of mechanical ventilation was five days (range: 2–7). No therapies other than antibiotics ((piperacillin/tazobactam + azithromycin or levofloxacin) for seven days) and systemic corticosteroids for COVID‐19 were used. Venous thromboembolism prophylaxis was given to all patients by subcutaneous injection of unfractionated heparin. Secondary infections related to corticosteroid use were not observed. Side effects included hyperglycaemia in five patients and delusion in two patients. Table 1 and Figure 1 show the clinical features and timeline of cases. Figure 2 shows unenhanced computed tomography images of each patient on admission day.

**Table 1:** Clinical features of cases.

	Case 1	Case 2	Case 3	Case 4	Case 5	Case 6	Case 7
Age	72	49	77	57	71	41	69
Sex	Male	Male	Female	Female	Male	Male	Female
BMI	22.7	25.1	19.6	30.8	21.2	31.7	25.5
Brinkman index	1600	480	150	Never	Never	40	Never
Initial symptoms	Fever, stomach ache	Fever, diarrhoea	Fever, loss of appetite	Fever, cough	Fever, cough	Fever, cough	Fever, dyspnoea
Chest CT findings on admission	Multiple GGO in bilateral lungs	Non‐segmental, patchy GGO in bilateral lungs	GGO of peripheral predominance in bilateral lungs	Non‐segmental, patchy GGO in peripheral predominance of bilateral lungs	GGO and consolidation in the bilateral inferior lungs	Multiple panlobular consolidation with fine reticular opacities, vascular thickening	Widespread GGO in bilateral lungs, mainly subpleural
Comorbidities	None	Asthma	Diabetes, hypertension, dyslipidaemia	Diabetes, hypertension, dyslipidaemia	Chronic atrial fibrillation	Diabetes, hypertension, asthma	None
Other respiratory pathogen infection	None	None	None	None	None	None	None
PaO_2_/FiO_2_ ratio before intubation	114	156	100	117	76	140	133
PaO_2_/FiO_2_ ratio after intubation	182	170	108	127	120	160	142
FiO_2_ at 24 h after intubation	0.4	0.4	0.4	0.35	0.45	0.35	0.4
Corticosteroid therapy	mPSL 1000 mg × three days followed by 1 mg/kg/day	mPSL 1000 mg × three days followed by 1 mg/kg/day	mPSL 1000 mg × three days followed by 1 mg/kg/day	mPSL 1000 mg × three days followed by 1 mg/kg/day	mPSL 500 mg × three days followed by 1 mg/kg/day	mPSL 500 mg ×three days followed by 1 mg/kg/day	mPSL 500 mg × three days followed by 1 mg/kg/day
Corticosteroid period (days)	13	16	15	13	11	13	11
Admission to intubation (days)	6	1	0	1	6	0	0
Initial symptoms to intubation (days)	11	10	11	12	12	4	13
Intubation period (days)	2	7	7	3	5	5	4
Clinical outcomes	Improved	Improved	Improved	Improved	Improved	Improved	Improved
RT‐PCR positive to negative (days)	16	14	13	13	14	23	14

BMI, body mass index; CT, computed tomography; FiO_2_, fraction of inspired oxygen; GGO, ground‐glass opacity; mPSL, methylprednisolone; PaO_2_, partial pressure of arterial oxygen; RT‐PCR, real‐time polymerase chain reaction.

## Discussion

Herein, we report a series of seven mechanically ventilated patients with ARDS‐related COVID‐19 who were administered early high‐dose, short‐term corticosteroid therapy.

Early studies have shown that increased amounts of serum proinflammatory cytokines were associated with pulmonary inflammation and extensive lung damage in patients with SARS‐CoV and Middle East respiratory syndrome coronavirus (MERS‐CoV) infections [9, 10]. In COVID‐19, Huang et al. noted the cytokine storm to be associated with disease severity [1]. According to Yang et al. [2], the non‐survivors will likely expire within one to two weeks after ICU admission, and ARDS increases the risk of death. In view of the high levels of cytokines induced during SARS‐CoV, MERS‐CoV, and SARS‐CoV‐2 infections, corticosteroids have been used frequently for the treatment of severe illness, for the possible benefit of reducing inflammation‐induced lung injury. However, current evidence in patients with SARS and MERS suggests that corticosteroids had no effect on mortality, but instead delayed viral clearance [11, 12]. Therefore, glucocorticoids should not be routinely administered to patients with COVID‐19, unless there is a separate evidence‐based indication (e.g. asthma or chronic obstructive lung disease exacerbation, refractory septic shock, and adrenal insufficiency) [13]. However, administration of glucocorticoids in critically ill patients with COVID‐19‐related ARDS is controversial. Wu et al. reported a retrospective cohort analysis of patients with COVID‐19 who had developed ARDS [5]. They revealed methylprednisolone treatment to be associated with decreased risk of death (hazard ratio: 0.38; 95% CI: 0.20–0.72). Furthermore, Villar et al. noted that early administration of dexamethasone could reduce duration of mechanical ventilation and overall mortality in patients with severe ARDS [14].

We hypothesized that high‐dose corticosteroid therapy could prevent tissue damage, thereby mitigating the degree of lung injury. We began high‐dose corticosteroid therapy early in the process of respiratory failure before any progression of viral pneumonia‐related ARDS. As some reports of corticosteroid therapy for SARS and MERS indicated that such a treatment could be damaging and worsen patient prognosis [15, 16], we limited our patients to short‐term regimens. The initiation of methylprednisolone intravenously reduced patients' fever and led to weaning from mechanical ventilation. As a result, a 100% survival rate was achieved, and reintubation rates were 0%, followed by complete withdrawal of ventilator support in all cases within seven days.

The findings from this case series suggest that high‐dose, short‐term corticosteroid therapy early in respiratory failure may provide a good prognosis of patients with COVID‐19‐related ARDS without critical side effects of corticosteroids. This study is a single‐centre report and the number of cases is limited. Further studies are required to clarify the effect of corticosteroid treatment in COVID‐19.

### Disclosure Statement

Appropriate written informed consent was obtained for publication of this case series and accompanying images.

At the time when this report was accepted for publication, the authors declared that the patients in this report had not been included in any previously published report on COVID‐19 that they had authored.
